# Knowledge and Attitude on Eye Donation Among Medical and Nursing Students in a Tertiary Care Centre in Western India

**DOI:** 10.7759/cureus.79117

**Published:** 2025-02-16

**Authors:** Afrid T Jaipuri, Sanjeevani V Ambekar

**Affiliations:** 1 Ophthalmology, Byramjee Jeejeebhoy Government Medical College and Sassoon General Hospital, Pune, IND

**Keywords:** attitude, corneal transplant, cross-sectional study, eye donation, india, keratoplasty, knowledge, medical students, nursing students, questionnaire

## Abstract

Background

Corneal blindness poses a significant public health concern in India. Penetrating keratoplasty stands out as the most efficient and frequently conducted solid organ transplantation procedure, providing significant potential for visual rehabilitation in affected patients, but its broad application continues to be impeded by the limited availability of donor corneas. The low rate of eye donation has been attributed to both the reluctance of potential donors to pledge their eyes and the inadequate efforts by healthcare workers to educate the public about the eye donation process, signifying the need to assess the knowledge and attitude about eye donation among medical and nursing students.

Purpose

This study aimed to assess the knowledge and attitude about eye donation among medical and nursing students in a tertiary care centre in Western India.

Methodology

In this cross-sectional, descriptive study, 500 medical and nursing students were selected as participants by convenience sampling. Knowledge and attitude were assessed regarding eye donation by using a self-designed, pre-validated questionnaire, which contained 13 questions (eight in the knowledge domain and five in the attitude domain). Descriptive statistics were used to elaborate on the participants’ demographic characteristics. The categorical variable was measured in percentage while the continuous variable was conveyed as mean ± SD. A p-value <0.05 was considered statistically significant. All analysis was performed using IBM SPSS Statistics for Windows, version 20.

Results

Out of 500 participants who completed the questionnaire, 204 (40.8%) were male and 296 (59.2%) were female. It was seen that 47 participants (9.4%) had excellent knowledge (score > 6), 355 participants (71%) had good knowledge (score 4-6), and 98 participants (19.6%) had poor knowledge (score < 4) about eye donation. Only 245 participants (49%) were willing to donate their eyes.

Conclusion

There is a need to impart knowledge and modify attitudes about eye donation among medical and nursing students since they will be playing a major role in educating and motivating potential donors regarding eye donation. Mass education and awareness programs are also needed to encourage the attitude of a family as a unit since many of the participants were hesitant to donate because of a lack of support from family.

## Introduction

Corneal blindness poses a significant public health concern in India, affecting an estimated 0.46% of the adult population [1]. This condition, which could have been remedied through corneal transplantation, underscores the urgency for enhanced awareness and access to appropriate medical interventions [1,2]. Major contributors to corneal blindness among adults living in developing nations include corneal scars (28.1%) and active keratitis (12.2%). Similarly, in the pediatric age group, acquired non-traumatic scars (71.32%) have been identified as the predominant indication for keratoplasty in developing regions [3]. Corneal transplantation offers a viable solution for many individuals currently suffering from corneal diseases and blindness. Penetrating keratoplasty stands out as the most efficient and frequently conducted solid organ transplantation procedure, providing significant potential for visual rehabilitation in affected patients [4]. Although there have been remarkable advancements in instrumentation, surgical methodologies, and perioperative care related to keratoplasty, its broad application continues to be impeded by the limited availability of donor corneas, a challenge prevalent in both developed and developing nations [5]. The data on eye donation in India highlights a significant and continuously expanding backlog of cornea transplantation needs. Research reveals that only a mere 0.5% of all deaths (1.2 million deaths in 2013-2014) resulted in the eyes being collected for transplantation purposes [6]. The low rate of eye donation has been attributed to both the reluctance of potential donors to pledge their eyes and the inadequate efforts by healthcare workers to educate the public about the eye donation process [5].

Eye donation involves the act of donating one's eyes after death. It is important to note that only corneal blindness can be addressed through this process, not other forms of blindness. However, there exists a substantial disparity between the demand and supply of corneas primarily due to low awareness. According to the Eye Bank Association of India, the current annual cornea procurement rate in India stands at 22,000 per year [6]. It is estimated that a considerable portion of donor corneas are deemed unsuitable for transplantation. Given our current ratio of available safe donor eyes, to perform 100,000 corneal transplants annually in India, we would require approximately 277,000 donor eyes [7]. The Hospital Corneal Retrieval Programme serves as a primary strategy for collecting donated eyes, focusing on motivating the relatives of terminally ill patients and accident victims to consider eye donation [6]. Hence, it is of utmost importance to assess the knowledge and attitude about corneal transplantation among healthcare workers since they play a major role in educating and motivating potential donors regarding eye donation [1,8-10].

## Materials and methods

Study design

A cross-sectional study using the convenience sampling method was conducted among medical and nursing students.

Study setting

The study was conducted in Byramjee Jeejeebhoy Government Medical College and Sassoon General Hospital, a tertiary care centre in Pune, Western India, among medical (71.2%) and nursing (28.8%) students.

Sample size

A self-designed questionnaire was distributed to 500 participants who were willing to participate in the study (see Appendix). Postgraduate residents working in all departments (pre-clinical, para-clinical, and clinical), students studying in the first year to the final year of MBBS, interns, and nursing students were included in the study. Postgraduate residents working in the Ophthalmology Department were excluded from the study.

Study tool

A self-designed, pre-validated questionnaire was used, which contained questions to assess the knowledge and attitude about eye donation. The questionnaire was divided into the following: 1) Demographic part: questions regarding age, sex and education status; 2) Knowledge domain: eight questions to test the knowledge of the participants; and 3) Attitude domain: five questions to test the attitude of the participants.

Validity

The questionnaire was validated by five independent subject experts. The questionnaire underwent a pilot test involving 10 medical students and 10 nursing students who were not involved in the study and had no prior knowledge about the topic. This pilot test aimed to assess the questionnaire's clarity and reliability (Cronbach's alpha = 0.82). Data collection began only after confirming that the survey instrument was readable, valid, and clear. This study was conducted after the approval of the Institutional Ethics Committee of Byramjee Jeejeebhoy Government Medical College and Sassoon General Hospitals, Pune (approval number: BJGMC/IEC/Pharmac/ND-Dept. 0923283-283).

Study procedure

The data were collected from medical and nursing students in a tertiary care centre in Western India. The participants were asked to fill out the questionnaire after obtaining informed written consent from them.

The knowledge section had multiple-choice questions. The participants received one point for each correct answer, while incorrect answers were scored as zero. Knowledge of the participants was assessed based on their total score, which ranged from zero to eight. A score of more than six was considered 'excellent', a score of four to six was considered 'good', and a score of less than four was considered 'poor'. Questions in the attitude section were open-ended. Responses in the attitude section were recorded as percentages.

Statistical analysis

The data was computed and analyzed by using IBM SPSS Statistics for Windows, Version 20.0 (released 2011, IBM Corp., Armonk, NY), and descriptive analyses were conducted. The results for each item on the questionnaire were reported as frequencies and percentages. Mean knowledge scores were compared for some of the demographic characteristics like gender and educational status. P-values <0.05 were considered significant.

## Results

Demographic characteristics

Five hundred medical and nursing students participated in the survey. As shown in Table 1, the majority of the respondents are females (296, 59.2%), whereas 204 (40.8%) were males. 

**Table 1:** Gender distribution

Gender	Frequency	Percentage
Male	204	40.8%
Female	296	59.2%

The mean age of the study participants was 22.704 years, as shown in Table 2 (range: 18-30 years, standard deviation: 2.84). 

**Table 2:** Age distribution

Age (years)	18	19	20	21	22	23	24	25	26	27	28	29	30
Frequency	8	33	76	90	63	105	9	8	34	24	34	10	6
Percentage	1.6	6.6	15.2	18	12.6	21	1.8	1.6	6.8	4.8	6.8	2	1.2

**Figure 1:**
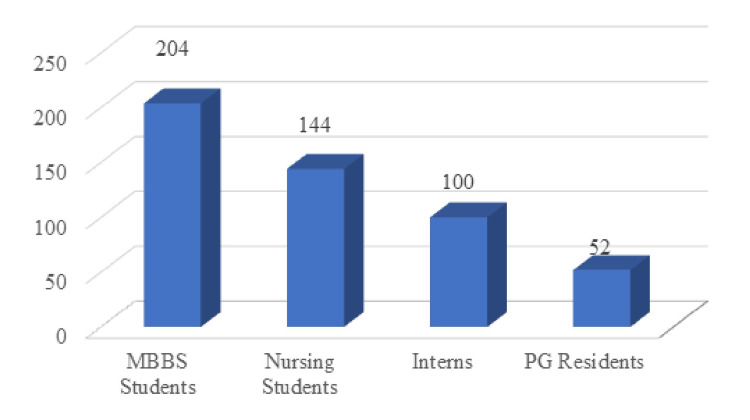
Educational status MBBS: Bachelor of Medicine, Bachelor of Surgery; PG: postgraduate

Two-hundred-four (40.8%) participants were MBBS students, and 144 (28.8%) participants were nursing students. Interns (20%) and postgraduate residents (10.4%) comprised the remaining sample size, as shown in Figure 1. All figures were made in Microsoft Excel (Microsoft Corp., WA, USA).

Question responses

According to Table 3, a staggering 408 (81.6%) participants knew that only the cornea of the donated eye could be used for transplantation, but only 176 (35.2%) knew that either the cornea or the entire eyeball could be removed during the donation process. Two-hundred twenty-seven (45.4%) were misinformed that the entire eyeball needs to be removed during the donation process, and 91 (18.2%) thought that cornea removal is the only way for eye donation. The majority of the participants (354, 70.8%) knew that the cornea should ideally be removed within six hours post death declaration, and 121 (24.2%) thought that we could wait till 24 hours for donation. Two-hundred sixty-two (52.4%) were unaware that it is not possible to specify who will receive the donated eye. Moreover, 324 (64.8%) correctly knew that facial appearance is not altered during eye donation, whereas 152 (30.4%) thought that eye donation would alter their facial appearance, which is one of the leading reasons for the unwillingness to eye donation. The study shows that 308 (61.6%) knew that HIV and hepatitis B and C are contraindications for eye donation. Only 187 (37.4%) knew that either Cornisol or MK medium could be used for preserving the donated eye. It was seen that 318 (63.6%) knew that the age limit is decided by the eye bank standard operating protocol and there is no fixed age limit across the nation.

**Table 3:** Knowledge about eye donation HIV: human immunodeficiency virus, MK Medium: McCarey-Kaufman, SOP: standard operating procedure

	Questions	Options	Responses (percentage)
1)	Which part of the eye is removed?	Cornea	91 (18.2%)
		Entire eyeball	227 (45.4%)
		Entire eyeball or Cornea (correct answer)	176 (35.2%)
		Don’t know	6 (1.2%)
2)	Which part of the eye is utilized?	Cornea (correct answer)	408 (81.6%)
		Entire eyeball	66 (13.2%)
		Don’t know	26 (5.2%)
3)	Cornea should be ideally removed within how many hours post death declaration?	6 hours (correct answer)	354 (70.8%)
		24 hours	121 (24.2%)
		Don’t know	25 (5%)
4)	Is it possible to specify who will receive the donated eye?	Yes	191 (38.2)
		No (correct answer)	238 (47.6%)
		Don’t know	71 (14.2%)
5)	Is the facial appearance altered after eye donation?	Yes	152 (30.4%)
		No (correct answer)	324 (64.8%)
		Don’t know	24 (4.8%)
6)	Which of the following is/are a contraindication for eye donation?	HIV	78 (15.6%)
		Hepatitis B/C	42 (8.4%)
		Both A & B (correct answer)	308 (61.6%)
		Don’t Know	72 (14.4%)
7)	Donated eye is preserved in which medium?	Cornisol	102 (20.4%)
		MK Medium	101 (20.2%)
		Either A or B (correct answer)	187 (37.4%)
		Don’t know	110 (22%)
8)	What is the age limit to donate eyes?	55 years	64 (12.8%)
		65 years	63 (12.6%)
		Decided by the Eye bank SOP (correct answer)	318 (63.6%)
		Don’t know	55 (11%)

Knowledge domain

**Figure 2:**
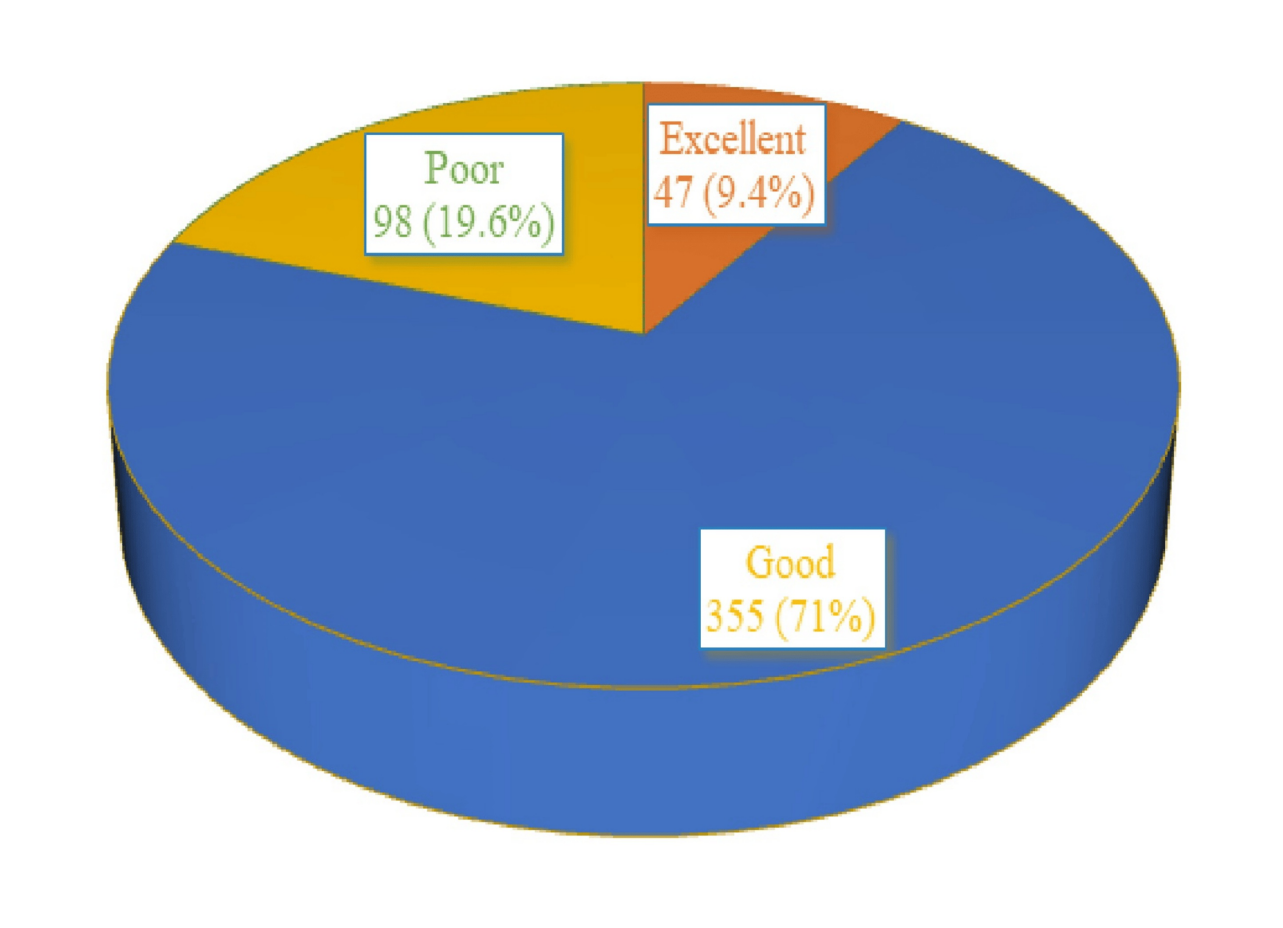
Knowledge domain

As shown in Figure 2, the knowledge domain was assessed; there were eight questions and every correct answer was given one mark. A score of >6 was considered 'excellent', a score of 4-6 was considered 'good', and a score of <4 was considered 'poor'. Only 9.4% (47) of the participants had excellent knowledge about eye donation, whereas 19.6% (98) of the participants had poor knowledge of the subject. The remaining 71% (355) had good knowledge about eye donation.

Attitude domain

**Figure 3:**
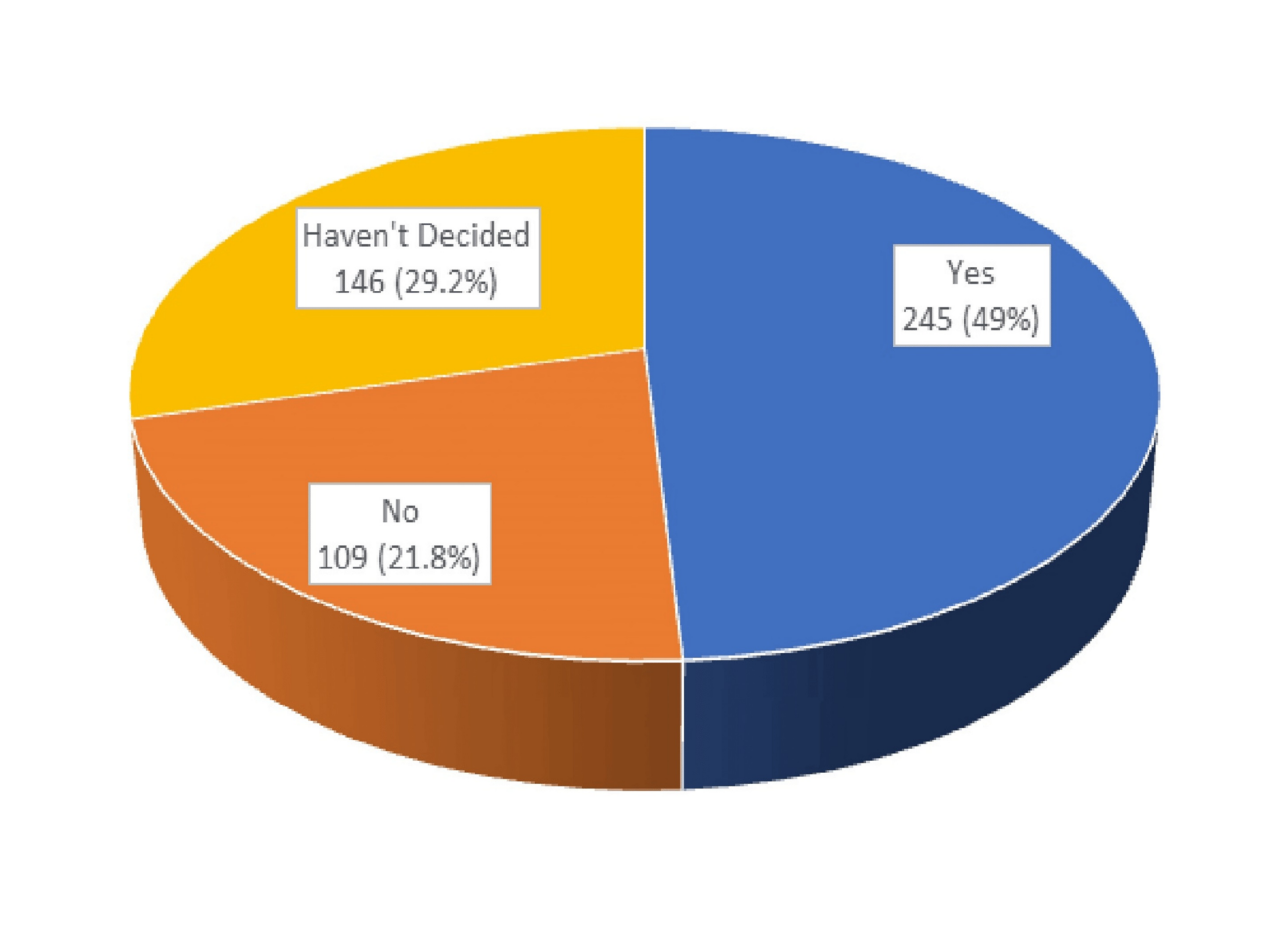
Willingness to donate eyes

**Figure 4:**
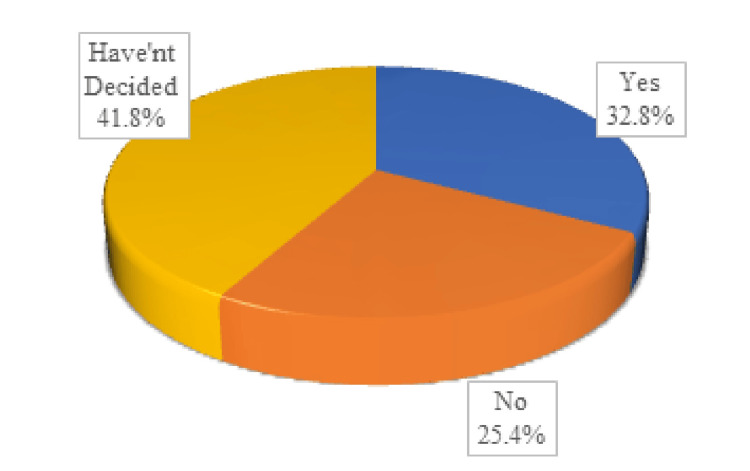
Willingness to donate a relative's eyes

**Figure 5:**
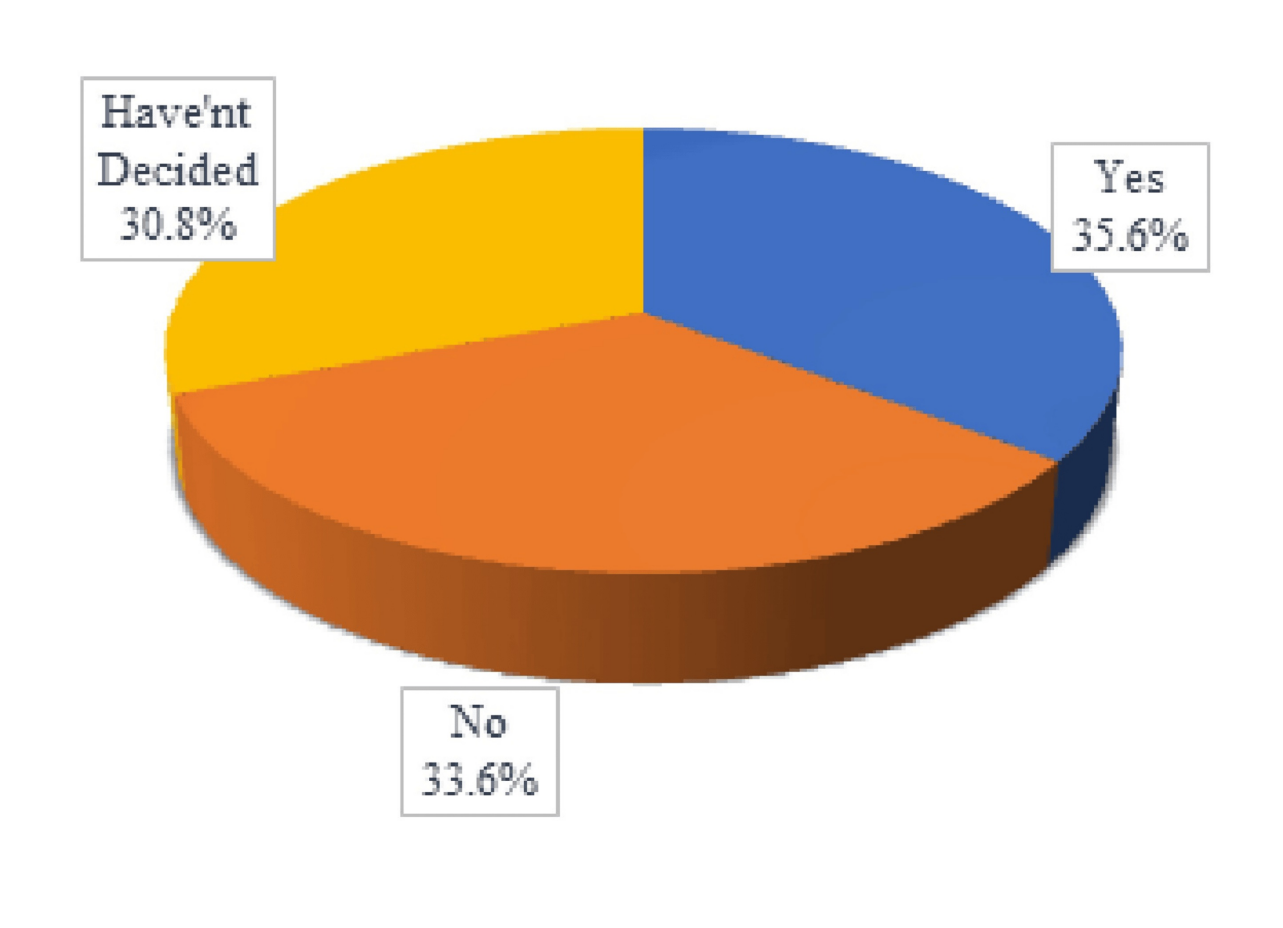
Willingness to donate eyes for research/training purposes

The attitude domain of the questionnaire included five questions regarding willingness for eye donation. According to Figure 3, the majority of the respondents (49%) were willing to donate their own eyes. Only 32.8% were willing to donate their relative’s eyes, and only 35.6% were willing to donate eyes for research/ training purposes, as shown in Figures 4-5.

**Figure 6:**
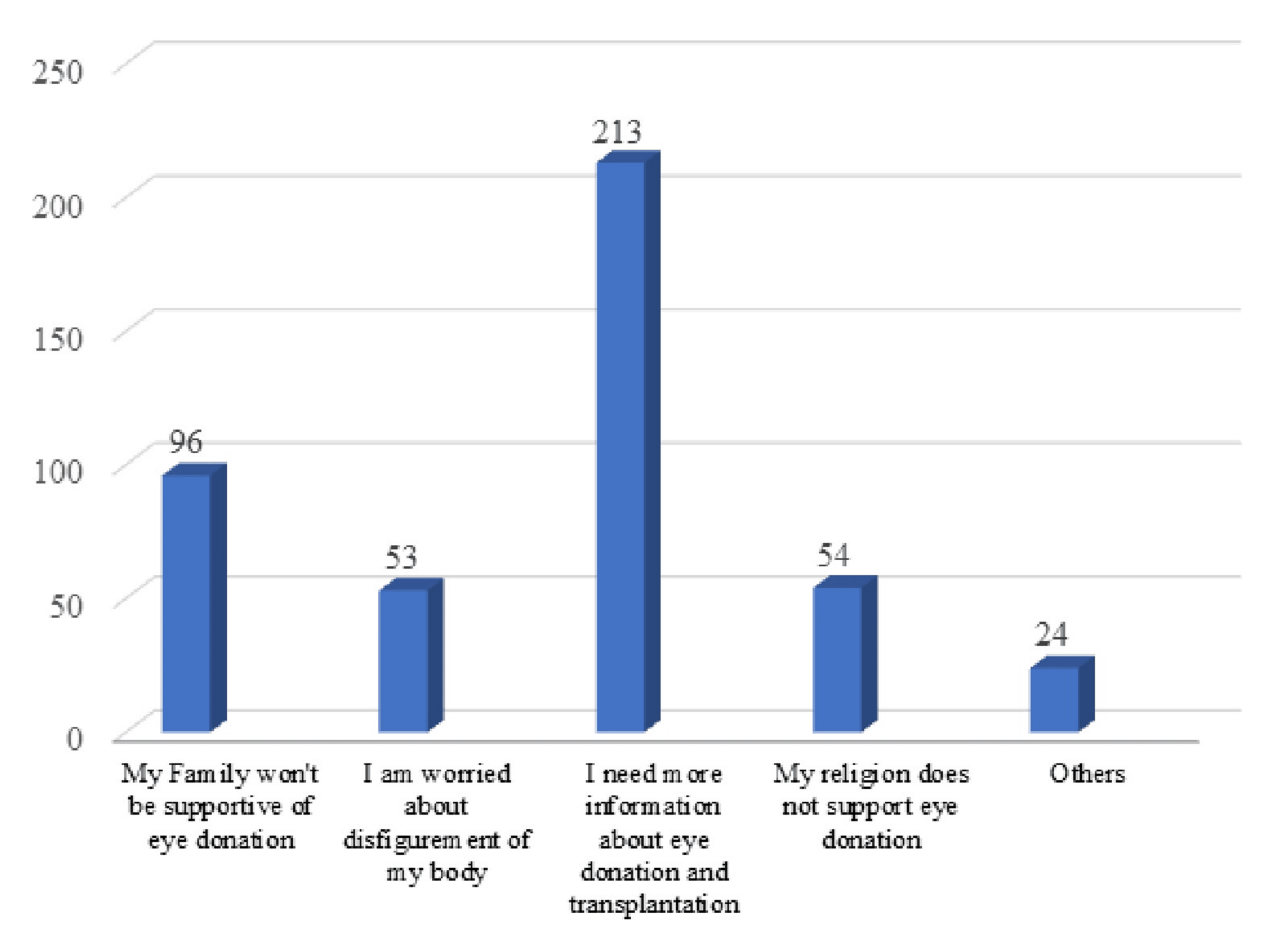
Reasons for unwillingness/undecidedness for donating eyes

As shown in Figure 6, the most common reason cited for unwillingness/undecidedness for donating eyes was 'I need more information about eye donation and transplantation'. It was followed by the reasons 'My family won’t be supportive of eye donation', 'My religion does not support eye donation', and 'I am worried about the disfigurement of my body'.

**Figure 7:**
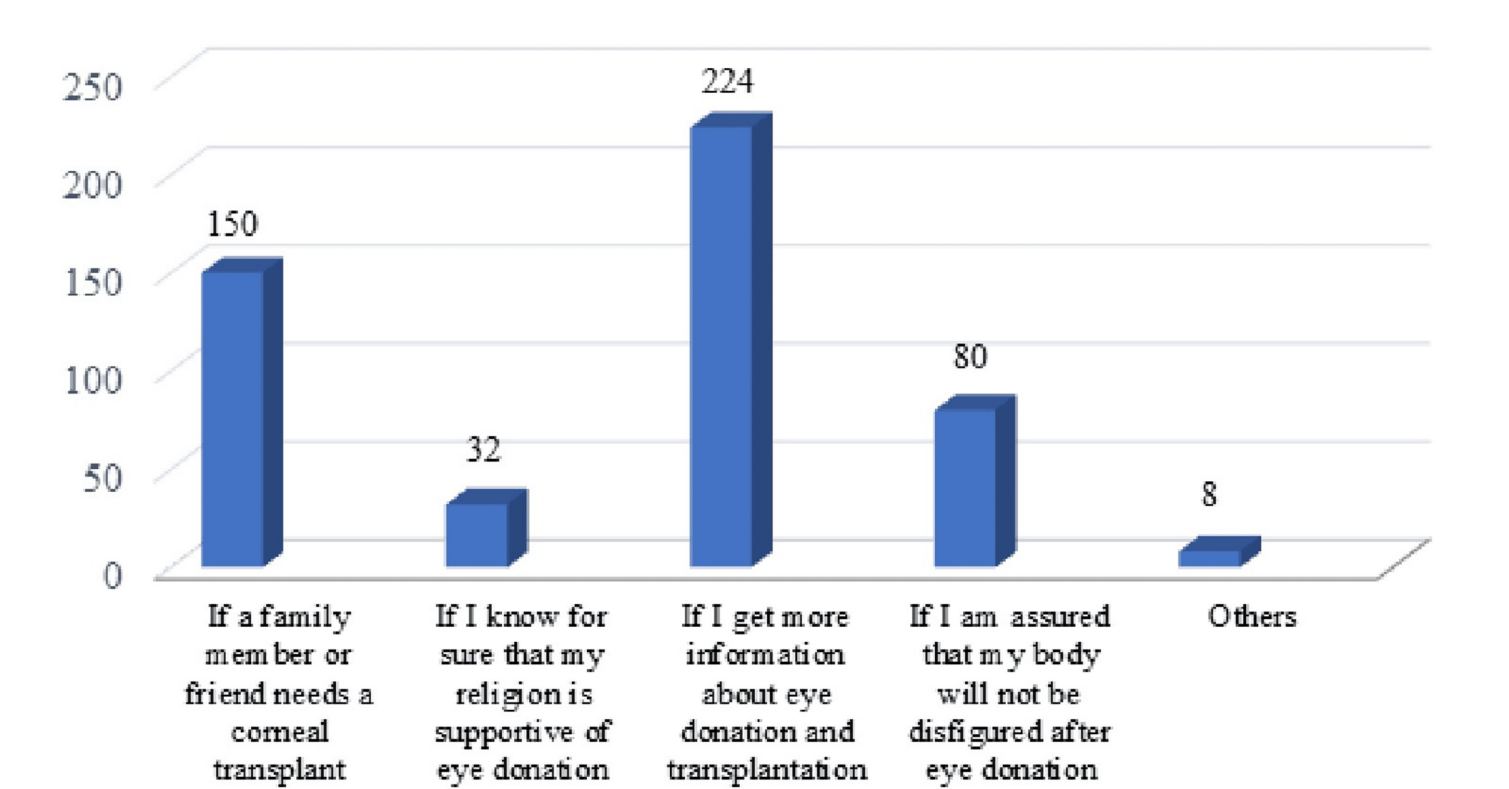
Motivating factors toward eye donation

Figure 7 shows that most participants selected 'If I get more information about eye donation and transplantation process' as the motivating factor toward eye donation, which was followed by 'If a family member or friend needs a corneal transplant', 'If I am assured that my body will not be disfigured after eye donation', and 'If I know for sure that my religion is supportive of eye donation'.

Comparison of the mean knowledge score

According to Table 4, a comparison of the mean knowledge score was performed, and it was found there is a statistical difference among genders (p = 0.002698) and also between medical and nursing students (p < 0.0000001). The mean knowledge score of females (4.793919) was better than their male colleagues (4.382353). Similarly, the mean knowledge scores were better among medical students (5.564607) than the nursing students (4.277778).

**Table 4:** Comparison of the mean scores Statistical test used: unpaired t-test

Demographics		Frequency	Mean knowledge score	Standard deviation	P-value
Gender	Females	296	4.793919	1.409927	0.002698
	Males	204	4.382353	1.556296	
Education status	Medical students	356	5.564607	1.318892	<0.0000001
	Nursing students	144	4.277778	1.371256	

Comparison of willingness for eye donation

According to Table 5, a comparison of willingness for eye donation was done among males and females. There was no statistically significant difference (p = 0.1156) between the willingness for eye donation among the two groups. However, when we compared the willingness for eye donation among medical and nursing students, the results showed a statistically significant difference (p = 0.002405) in the attitude toward eye donation.

**Table 5:** Comparison of willingness for eye donation Statistical test used: chi-squared test

Demographics		Willing	Not willing/not decided	P-value
Gender	Females	153	143	0.1156
	Males	90	114	
Education status	Medical students	213	143	0.002405
	Nursing students	64	80	

## Discussion

The late Dr. Muthiah started the very first eye bank in India, and he successfully performed the first corneal transplant in 1948 [11]. Seventy-six years down the line, there is still a huge backlog of corneas for corneal transplantation as patients keep waiting for donated corneas. Educating the masses about eye donation serves as the strongest weapon in increasing the supply of donated corneas amidst the ever-increasing demand. Around 18.7 million individuals are estimated to be blind in India [12] and 190,000 individuals are blind due to bilateral corneal diseases. Every year, an additional 20,000 individuals are added to the list [13]. The study by Tontus et al. emphasized the significance of educating healthcare workers, who serve as frontline personnel capable of fostering trust, motivating potential donors and their families, and educating the public regarding various forms of organ donation [14]. Medical and nursing students are the future frontline soldiers in this battle for procuring corneas for corneal blind patients.

In our study, it was seen that the majority (80.4%) of the students had excellent and good knowledge about eye donation. The knowledge about eye donation was found to be statistically better among medical students than nursing students (p = 0.002405). According to the study conducted by Dhaliwal et al. in 2002, 79.6% of medical students demonstrated a good awareness regarding corneal donation [15]. Yadav et al. reported that nursing students also exhibited a good level of knowledge about eye donation and its process [16]. Ahirwar RK et al. concluded that 81.4% of medical students had a good awareness of eye donation [17]. Kumar S et al. also found that awareness and knowledge about eye donation were statistically better than nursing and paramedical students in Bhopal [18].

Despite such positive numbers in knowledge and awareness about eye donation among medical and nursing students, the increased knowledge did not necessarily convert into increased willingness and pledge for eye donation. This study found that only 59.83% of medical students were willing to donate their eyes. The numbers were even lower for nursing students at 44.44%, a statistically significant (p < 0.0000001) difference between medical and nursing students, which corresponds to the statistically significant (p = 0.002405) difference in knowledge among the two groups. However, such a difference in willingness (p = 0.1156) for eye donation could not be correlated to the difference in knowledge (p = 0.002698) between the two genders. Srivastava et al. studied the willingness for eye donation among first- and second-year MBBS students, which was a commendable 83.79% [19]. The number was just 61.9% when Ahirwar et al. recorded the willingness among medical students [17]. The numbers were 88.4% among medical students and 79.1% among nursing students when Chowdhary et al. recorded the willingness for eye donation in 2018 [20]. Only 32.8% of the participants in this study were willing to donate a relative’s eyes and only 35.6% were willing to donate eyes for research or training purposes.

The lack of information was cited as the most common reason for unwillingness to eye donation. The lack of family support and religion were the other hindrances for eye donation according to the participants. Most participants felt that getting more information would motivate them for eye donations. Concerns were also raised about the disfigurement of the face after the donation process which needs to be addressed to motivate more people to donate. According to the study conducted by Chowdhary et al., lack of awareness and objections from family members were perceived as the most common reasons for non-donation among medical and nursing students [20]. Similar reasons were cited for non-donation by nursing students in the study by Gupta et al [21].

Since 1986, India has observed the Eye Donation Fortnight annually from August 25 to September 8. This initiative was established following the late Prime Minister of India, Shri Rajiv Gandhi's pledge to donate his eyes on his birthday, which falls on August 25. The celebration aims at spreading awareness about eye donation among the general public.

Limitations

The study was conducted at a single tertiary care centre. In order to generalise the result, other centres should also be included in the study. Also, the self-reported questionnaire may introduce response bias in the results. Other healthcare professionals should also be included in the study to get a broader understanding of the healthcare community's knowledge and attitude towards eye donation.

## Conclusions

Awareness and knowledge about eye donation were found to be better among medical students than nursing students according to this study. The majority of the participants had good knowledge about eye donation, but willingness to donate was reported by less than half. These students are going to be future doctors and nurses and will be playing a crucial role in motivating the masses for eye donation. Hence, it is of utmost importance to educate these students about eye donation and motivate them for eye donation, so that they can in turn educate the general public and in turn decrease the long-standing backlog of eye donation by motivating the masses for eye donation. It is also important to note that just educating the students is not enough. There is also a need to encourage the attitude of ‘family as a unit’ since the lack of family support was cited by the participants as a major hindrance for eye donation.

## Appendices

Study questionnaire

Knowledge

1) Which part of the eye is removed? A) Entire eyeball B) Cornea

C) Either A or B

D) Don’t know 2) Which part of the eye is utilized? A) Entire eyeball B) Cornea C) Don't know 3) Cornea should be ideally removed within how many hours post death certification? A) 6 hours B) 24 hours C) Don't know 4) Is it possible to specify who will receive the donated eye? A) Yes B) No C) Don't know 5) Is the facial appearance altered after eye donation? A) Yes B) No C) Don't know 6) Which of the following is a contraindication for eye donation? A) HIV B) Hepatitis B/C C) Both A & B D) Don't know

7) Donated eye is preserved in which medium? A) Cornisol B) MK Medium C) Either A or B D) Don't know 8) What is the age limit to donate? A) 55 years B) 65 years C) Decided by the Eye Bank Standard Operating Protocols D) Don't know

Attitude

1) Are you willing to donate your eyes? A) Yes B) No C) Haven’t decided yet 2) Are you willing to donate your relative's eyes? A) Yes B) No C) Haven’t decided yet

3) Are you willing to donate eyes for research purposes?

A) Yes B) No C) Haven't decided yet

4) Reasons for unwillingness or undecidedness to donate your eyes? (Multiple choices allowed) A) My family won't be supportive of corneal donation B) I am worried about the disfigurement of my body C) I need more information about eye donation and transplantation D) My religion does not support corneal donation E) Other reasons (Please specify) -________________________________________________ 5) What will make you feel more positive about eye donation? (Multiple choices allowed) A) If my family member or friend needs a corneal transplant B) If I know for sure that my religion is supportive of corneal donation C) If I get more information about eye donation and the transplantation process D) If I am assured that my body will not be disfigured after eye donation E) Other reasons (Please specify) -________________________________________________
